# Equine Multiple Congenital Ocular Anomalies maps to a 4.9 megabase interval on horse chromosome 6

**DOI:** 10.1186/1471-2156-9-88

**Published:** 2008-12-19

**Authors:** Lisa S Andersson, Rytis Juras, David T Ramsey, Jessica Eason-Butler, Susan Ewart, Gus Cothran, Gabriella Lindgren

**Affiliations:** 1 Dept of Animal Breeding and Genetics, Swedish University of Agricultural Sciences, Box 597, SE-751 24 Uppsala, Sweden; 2 Dept of Veterinary Integrative Biosciences, College of Veterinary Medicine and Biomedical Sciences, Texas A&M University, College Station, TX 77843-4458, USA; 3 Department of Small Animal Clinical Sciences, 240 National Food Safety and Toxicology Center, Michigan State University, East Lansing, MI 48824, USA; 4 Department of Large Animal Clinical Sciences, G-100 Veterinary Medical Center, Michigan State University, East Lansing, MI 48824, USA; 5 The Animal Ophthalmology Center, PLLC, 1300 W. Grand River Avenue, Williamston, MI 48895, USA; 6 Asterand, 440 Burroughs Detroit, MI 48202, USA

## Abstract

**Background:**

Equine Multiple Congenital Ocular Anomalies (MCOA) syndrome consists of a diverse set of abnormalities predominantly localized to the frontal part of the eye. The disease is in agreement with a codominant mode of inheritance in our horse material. Animals presumed to be heterozygous for the mutant allele have cysts originating from the temporal ciliary body, peripheral retina and/or iris. In contrast, animals predicted to be homozygous for the disease-causing allele possess a wide range of multiple abnormalities, including iridociliary and/or peripheral retinal cysts, iridocorneal angle abnormalities, cornea globosa, iris hypoplasia and congenital cataracts. MCOA is most common in the Rocky Mountain horse breed where it occurs at a high frequency among Silver colored horses. The Silver coat color is associated with mutations in *PMEL17 *that resides on ECA6q23. To map the *MCOA *locus we analyzed 11 genetic markers on ECA6q and herein describe a chromosome interval for the *MCOA *locus.

**Results:**

We performed linkage analysis within 17 paternal half-sib families of the Rocky Mountain horse breed. More than half of the 131 offspring had the Cyst phenotype and about one third had MCOA. Segregation data were obtained by genotyping 10 microsatellite markers most of which are positioned on ECA6q22-23, as well as the missense mutation for the Silver phenotype in *PMEL17*. Significant linkage was found between the *MCOA *locus and eight of the genetic markers, where marker *UPP5 *(Theta = 0, z = 12.3), *PMEL17ex11 *(Theta = 0, z = 19.0) and *UPP6 *(Theta = 0, z = 17.5) showed complete linkage with the *MCOA *locus. DNA sequencing of *PMEL17 *in affected and healthy control individuals did not reveal any additional mutations than the two mutations associated with the Silver coat color.

**Conclusion:**

The *MCOA *locus can with high confidence be positioned within a 4.9 megabase (Mb) interval on ECA6q. The genotype data on *UPP5*, *PMEL17ex11 *and *UPP6 *strongly support the hypothesis that horses with the Cyst phenotype are heterozygous for the mutant allele and that horses with the MCOA phenotype are homozygous for the mutant allele.

## Background

In 1999, Ramsey *et al*. [1] described a wide range of congenital ocular defects in the Rocky Mountain horse originally referred to as Anterior Segment Dysgenesis syndrome. More recently, the syndrome has been referred to as Multiple Congenital Ocular Anomalies (MCOA), as described by Grahn *et al*. [2]. Ocular abnormalities are in general quite rare in horses, accounting for less than five percent of the congenital disorders reported in horses [3,4]. Conversely, eye abnormalities were encountered in approximately half of the 514 horses examined in Ramsey's study. The high incidence of ocular abnormalities in this breed is most likely due to a founder effect. Pedigree examination has revealed that a large proportion of the affected horses have a common ancestor, a stallion that is one of the few founders of the Rocky Mountain horse breed. The fact that five out of seven of the ancestral stallion's first-generation offspring had ocular abnormalities suggests that this individual did indeed carry the mutant allele. Extensive breeding from this foundation sire's offspring has propagated the causative mutation(s), leading to a high frequency of MCOA syndrome in the Rocky Mountain horse population. There are numerous examples where intense and selective breeding of a few lineages has led to a high occurrence of undesired traits in domesticated animals. Examples of such are hyperkalaemic periodic paralysis (HYPP) among Quarter Horses [5] and severe combined immunodeficiency disease (SCID) in Arabian horses [6,7]. The negative effects of breed development from a limited number of individuals and/or inbreeding is also evident in dogs, with several cases of high incidence of inherited diseases within modern purebreds [8].

**Figure 1:**
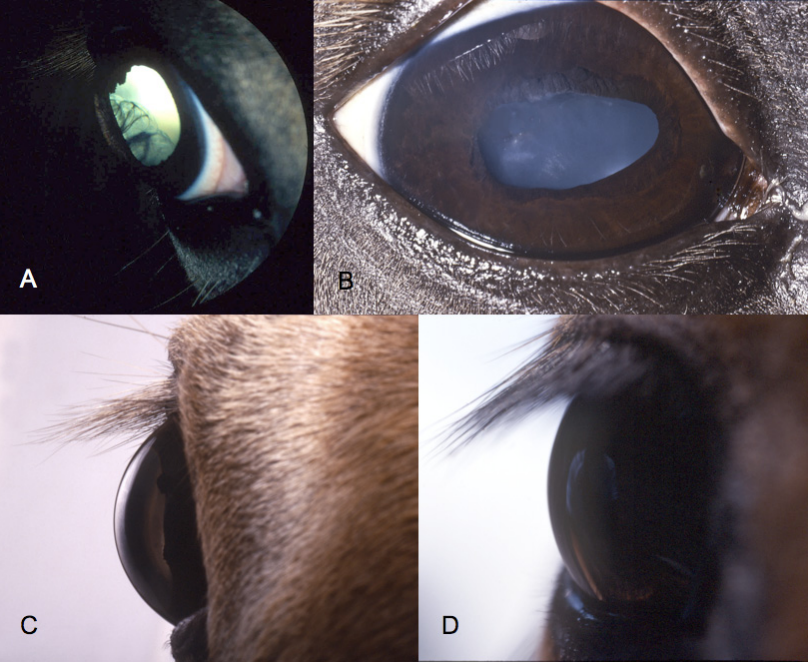
**Clinical signs of MCOA syndrome**. **A**. Oblique profile images of the lateral anterior segment of the right eye of a Rocky Mountain horse. A multiloculated cyst arising from the anterior ciliary body is present. **B**. Photograph of the right eye of a Rocky Mountain horse with ectropion uvea, dyscoria, cataract, and lens subluxation. The granula iridica is hypoplastic, the pupil is misshapen, and complete circumferential ectropion uvea is present. Nuclear cataract of the nuclear-cortical junction is present. Vitreous is present in the anterior chamber between the iris and lens secondary to posterior ventral lens subluxation. **C**. Profile photograph of a Rocky Mountain Horse with Cornea Globosa. Note the anterior protrusion of the cornea and large corneal diameter. **D**. Profile photograph of a Rocky Mountain Horse with a normal cornea. Note the normal corneal curvature and diameter.

The MCOA syndrome has a codominant mode of inheritance [9] and the affected horses can be separated into two groups depending on the type of ocular defects that they possess; the Cyst phenotype and the MCOA phenotype. Horses referred to as having the Cyst phenotype are presumed to be heterozygous for the mutant allele and have a minor form of ocular abnormalities compared with horses carrying two copies [1,9]. The presumed heterozygous horses have cysts that originate from the temporal ciliary body, peripheral retina and/or iris (Figure 1a). A smaller number of these horses also have moderate retinal dysplasia or retinal detachment that appears to be an extension of these cysts [1]. The predicted homozygous horses have multiple abnormalities, primarily affecting the anterior segment of the eye (Figure 1b–d) [1,9]. They encompass all clinical signs included in the Cyst phenotype concurrent with iris hypoplasia, iridocorneal angle abnormalities, miosis, congenital cataracts, cornea globosa, iridocorneal adhesions and opacification, nuclear cataract as well as pupils with a decreased or absent light response and that do not dilate when administered mydriatic drugs. A single horse does not necessarily possess all of these clinical signs and there are additional defects detected less frequently [1,10].

**Figure 2:**
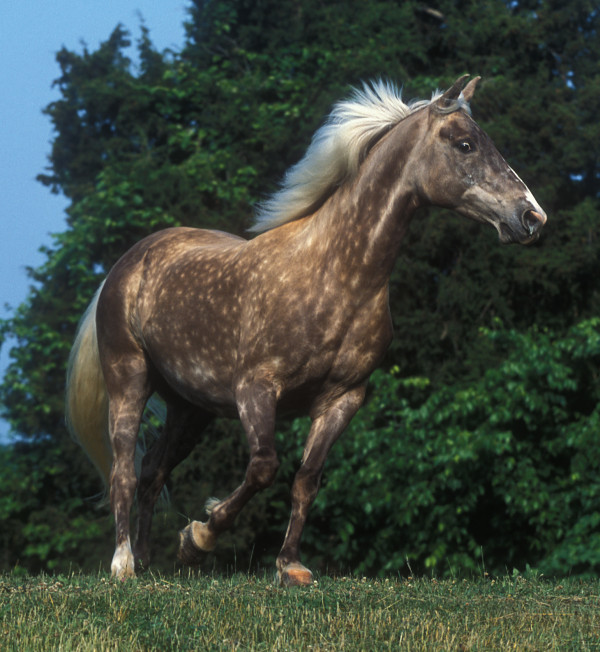
**A Silver colored Rocky Mountain Horse**. The typical shiny white mane and tail as well as a slightly diluted body color with dapples is seen in this genetically black Silver colored horse. The phenotype is caused by dilution of eumelanin in the hair to white or grey. The dilution is most visible in the long hairs of the mane and tail. The horse has also been diagnosed with MCOA.

MCOA has been well studied in a limited population of the Rocky Mountain horse breed, where it was found to be most frequent in horses with the Silver coat color (also known as Chocolate coat color) [1]. Silver colored horses are especially desired in this breed and thus used more frequently for breeding. The consequence is an unusually high frequency of Silver colored horses (approximately 55% of registered individuals are shown as Silver/Chocolate), as well as horses with MCOA syndrome. The percentage of Silver colored horses from different breeds that also display the Cyst phenotype or MCOA is unknown, simply because this has not been investigated. In many of the other horse breeds that have the Silver coat color, it is present in a much lower frequency. As a result most Silver colored horses are heterozygous for the *Silver *mutation and hence most likely carry the milder form of this eye disorder that is more difficult to detect. The investigation about the correlation between the Silver coat color and MCOA is further complicated by the fact that certain horse coat colors, for example chestnut (red), display no or little effect of the *Silver *mutation. The Silver color is characterized by dilution of black pigment in the hair to white or grey. The dilution is most visible in the long hairs of the mane and tail, where the dilution results in a silver or white mane and tail color (Figure 2). In 2006, Brunberg *et al*. [11] reported that the Silver coat color is associated with mutations in pre-melanosomal protein 17 (*PMEL17)*. This conclusion was based on the observation of no recombinants between *PMEL17 *and *Silver *in a pedigree and the identification of a haplotype, composed of sequence variations in intron 9 and exon 11, showing complete concordance with the presence of *Silver *across six different horse breeds. In that paper, we also describe a candidate causative missense mutation ARG618Cys that is a non-conservative substitution at a conserved site and mutations in the near vicinity cause a similar phenotype in chickens [12]. The intronic mutation was found less likely to be causative for Silver since it resides in an intronic region not well conserved among mammalian species. Interestingly, mutations in *PMEL17 *are known to give ocular abnormalities and visual impairment in the zebrafish mutant *fading vision *[13]. Microphthalmia and colobomas have also been described in merle patterned Dachshunds [14] and Australian Shepherds [15], a patterning associated with a SINE insertion in *PMEL17 *according to Clark *et al*. [16].

In this study we firmly positioned the *MCOA *locus to a 4.9 Mb region on ECA6q. Further, we describe complete linkage between three genetic markers in the interval and the *MCOA *locus.

## Results

### Genotyping and Linkage analysis

**Figure 3:**
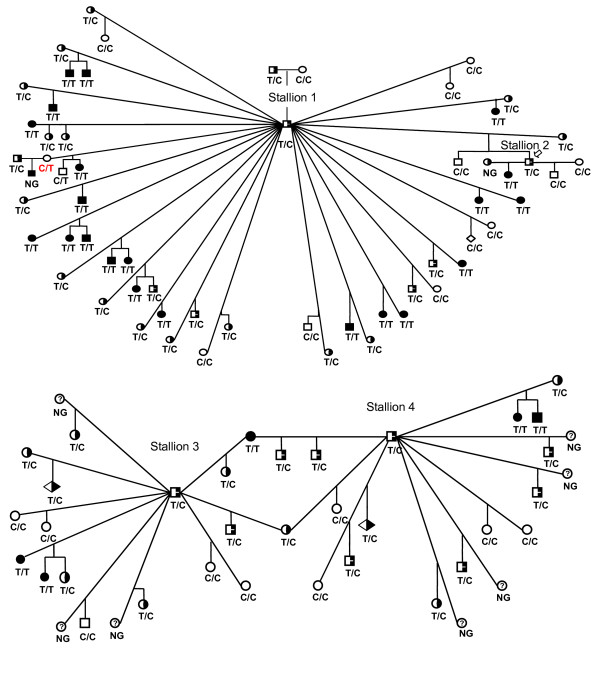
**Pedigrees**. Pedigrees of four of the half-sib families (sired by stallion 1–4) used in this study. Phenotype information along with genotype data on one genetic marker (*PMEL17ex11) *is shown for all individuals. Unaffected horses are indicated by open symbols, half filled symbols indicate horses affected with cysts, solid symbols indicate horses affected with MCOA and unexamined horses are indicated by symbols enclosing a question mark. We selected the SNP *PMEL17ex11 *to represent the near perfect correlation between phenotype and genotype of the three markers in our interval that show complete linkage with the *MCOA *locus. The only discrepancy (marked in red) is a healthy dam with a heterozygous genotype. However, since she has produced two MCOA affected offspring, this dam is most likely a case where the position and/or size of the cysts make them extremely difficult to detect. NG: Not Genotyped (as DNA was not available).

**Figure 4:**
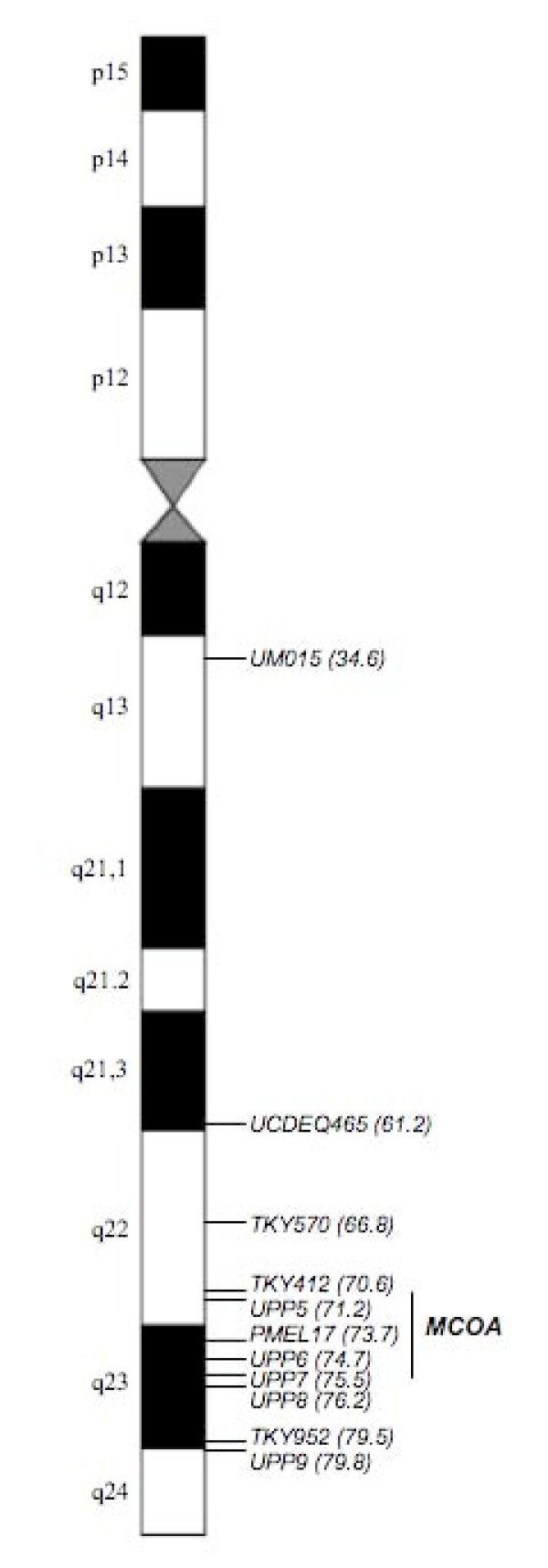
**Positions of analyzed ECA6q markers**. Ten microsatellite markers and one SNP marker (*PMEL17ex11*) spanning approximately 45.2 Mb were included in this study. Three markers showed complete linkage (recombination fraction = 0.00) with the *MCOA *locus; *UPP5 *(z = 10.84), *PMEL17ex11 *(z = 12.64), *UPP6 *(z = 11.14). Our result can position the MCOA susceptibility locus with high confidence to the 4.9 megabase interval between *TKY412 *and *UPP7*.

We performed linkage analysis by genotyping 17 paternal half-sib Rocky Mountain horse families segregating for the *MCOA *locus. The number of offspring sired from each stallion ranged from one to 29 with an average of 7.7 offspring per sire (Table 1). DNA was available from 41 out of the 94 different mares within the pedigree. We genotyped 11 genetic markers (Table 2), spanning approximately 45 Mb on ECA6q. The rationale for selecting this particular chromosome and region was that the *PMEL17 *locus is positioned on ECA6q23. Four families were used for identification of an initial and broader interval for the *MCOA *locus (Figure 3). Genotyping was restricted to cover a narrower region around *PMEL17 *for the remaining 13 families. For simplicity we only present the total number of informative meioses per marker, which is compiled in Table 3. According to CHROMPIC [17], one individual was a double recombinant over a very small genetic distance and was excluded from the analysis since it most likely represents a genotyping error. Interestingly, one offspring showed non-Mendelian inheritance for the *MCOA *locus. Although bred from a dam (marked red in Figure 3) without any detectable ocular abnormalities, this individual had the MCOA phenotype. The particular mare was, therefore, reported to be a case of nonpenetrance by Ewart *et al*. 2000 [9]. Genotyping revealed that this mare was heterozygous for all markers between *UCDEQ465 *and *UPP7 *(see Figure 4) and thus might represent a horse with a phenotyping error. This particular horse will be re-examined.

**Table 1:** Description of the pedigrees and extent of genotyping in each family

	**Number of offspring^1^**	**Number of different dams^2^**	**Number of offspring with dam genotype^3^**	**Number of markers**
Stallion 1	29 (15,8,6)	22 (5,12,5,0)	29 (7,16,6)	11
Stallion 2	2 (1,0,1)	2 (0,1,1,0)	2 (0,1,1)	11
Stallion 3	10 (1,6,3)	9 (2,2,2,3)	7 (3,2,2)	11
Stallion 4	12 (2,8,2)	9 (1,2,2,4)	8 (2,3,3)	11
Stallion 5	9 (1,5,3)	7 (0,1,0,6)	1 (0,1,0)	3–5
Stallion 6	12 (5,7,0)	10 (1,0,1,8)	2 (1,0,1)	3–5
Stallion 7	7 (2,4,1)	5 (0,0,0,5)	0 (0,0,0)	3–5
Stallion 8	19 (3,14,2)	15 (0,3,1,11)	4 (0,3,1)	3–5
Stallion 9	4 (3,1,0)	3 (1,2,0,0)	4 (1,3,0)	3–5
Stallion 10	4 (1,3,0)	4 (0,0,0,4)	0 (0,0,0)	3–5
Stallion 11	7 (2,4,1)	7 (0,3,0,4)	3 (0,3,0)	3–5
Stallion 12	5 (1,2,2)	5 (0,1,0,4)	1 (0,1,0)	3–5
Stallion 13	5 (0,4,1)	3 (0,0,0,3)	0 (0,0,0)	3–5
Stallion 14	1 (0,1,0)	1 (0,0,1,0)	1 (0,0,1)	3–5
Stallion 15	2 (1,1,0)	2 (1,0,0,1)	1 (1,0,0)	3–5
Stallion 16	1 (0,1,0)	1 (0,0,1,0)	1 (0,0,1)	3–5
Stallion 17	2 (0,1,1)	2 (0,1,0,1)	1 (0,1,0)	3–5

Total:	131		65	

^1^Phenotypes of the offspring are compiled within parenthesis (MCOA, Cyst, Healthy)

^2^Phenotypes of the dams are compiled within parenthesis (MCOA, Cyst, Healthy, Unexamined)

^3^Phenotypes of the dams are compiled within parenthesis (MCOA, Cyst, Healthy)

**Table 2:** Genetic markers used in the linkage analysis [18,27,28,11]

Marker	Position (bp) Ensembl [18]	Forward primer (5'-3')/ Reverse primer (5'-3')	Accession number/Reference
*UM015*	34558444	AGTCTGGCTGAGGATACTG GGTGAGAAAGGAGATAAATG	GenBank: AF195133 Chowdhary *et al. *[27]
*UCDEQ465*	61228896	AACCAGTCCCTACATAGAAC CTCACAACCAAGCATACA	GenBank: U67414 Chowdhary *et al. *[27]
*TKY570*	66793583	TCTCCGCAGCTCAAACTTTC CTCAAAGGTGCCTGAGAAGC	GenBank: AB103788 Tozaki *et al. *[28]
*TKY412*	70589360	GTGTGGGACAGGAAGTTTGG ATTCTTGGGTCCCCTCATCT	GenBank: AB103630 Tozaki *et al. *[28]
*UPP5*	71164435	GCACAGTCTAGGGGGTGTGT TCTGGGCCTGGGTAGGTAGT	Probe ID: 9710436 This study
Pyroseq. PCR primers-*PMEL17 *ex11 Pyroseq. Seq primer-*PMEL17 *ex11	73665164 73665164	Biotin-TCCATTGCTTACCAGTTTCCTT CTCACCAAAGGGGGAAGAG GCCCTGCTTCATAAGTCTG	GenBank: DQ855465 Brunberg *et al. *[11]
*UPP6*	74667027	ATTGTACCTGGGACCCTTCC TGTCTTTGCTTCCCAGTCCT	Probe ID: 9710437 This study
*UPP7*	75475262	CTTTGACACACCCTGGGAAG CATCCACATCCTTCATTTGCT	Probe ID: 9710438 This study
*UPP8*	76228607	TGTCCCTGAATTCTCTGATCC GACCTCCAGTCAAAATGAATCTG	Probe ID: 9710439 This study
*TKY952*	79472871	GATCGGTAAGTGTCGGGAC TAAAATGACTGGGTGGAGAC	GenBank: AB104170 Tozaki *et al. *[28]
*UPP9*	79806071	AAAAAGAGGATTGGCAACG TGTGTGGGGATGTAATTGGA	Probe ID: 9710440 This study

**Table 3:** Two-point analysis between the *MCOA *locus and ECA6q markers

**Marker**	**Recombination fraction** Θ	**Maximum LOD score** **Z_max_**	**Total number of informative meioses**
*MCOA – UM015*	0.50	0.00	22
*MCOA – UCDEQ465*	0.21	1.79	51
*MCOA – TKY570*	0.10	4.84	55
*MCOA – TKY412*	0.02	10.47	79
*MCOA – UPP5*	0.00	12.34	76
*MCOA – PMEL17ex11*	0.00	18.96	93
*MCOA – UPP6*	0.00	17.46	127
*MCOA – UPP7*	0.02	12.53	99
*MCOA – UPP8*	0.03	10.36	84
*MCOA – TKY952*	0.04	7.22	68
*MCOA – UPP9*	0.15	0.83	23

The results of the TWO-POINT analysis including all 17 paternal families are compiled in Table 3. We found complete linkage (Θ = 0.00) between the *MCOA *locus and the three markers *UPP5 *(z = 10.84), *PMEL17ex11 *(z = 12.64) and *UPP6 *(z = 11.14). Marker *UM015 *is approximately 27 Mb upstream of *UCDEQ465*, which explains the lack of linkage between this marker and the *MCOA *locus (see Figure 4). We used the BUILD option with a fixed order of markers according to the order of markers in Ensembl (EquCab 2, Sep 2007) [18] and allowed *MCOA *to be inserted. Since three markers did not have any recombination to the *MCOA *locus, the disease locus could be incorporated equally likely anywhere in the interval between *TKY412 *and *UPP7 *(log10_like = -25.24). According to the horse genome sequence in Ensembl, these two markers are positioned on Chr6:70589360 and Chr6:75475262, respectively. The current MCOA interval thus spans approximately 4.9 Mb. There is one reliable recombinant on either side of this interval.

### Sequencing of *PMEL17 *and genotyping of SNPs

*PMEL17 *is a candidate gene for MCOA because of its documented effect on ocular phenotypes in the zebrafish as well as described eye defects in merle-patterned dogs. In addition, the SNP marker positioned within the gene showed complete linkage to the *MCOA *locus in our linkage analysis. We therefore sequenced *PMEL17 *in three healthy Rocky Mountain horses, three horses with the MCOA phenotype and three with the Cyst phenotype. In total, 6400 bp was sequenced, covering all exons and UTRs, 370 bp upstream, 210 bp downstream as well as full coverage of all introns except four. No additional polymorphism that associated completely with the disease could be detected other than the two SNPs identified in Brunberg *et al*. 2006 [11]. In these two SNPs, all three MCOA horses were homozygous (Exon 11: T/T and Intron 9: T/T) for the alleles associated with the Silver coat color, the three healthy horses were homozygous (Exon 11: C/C and Intron 9: A/A) for the opposite alleles and the horses with the Cyst phenotype were heterozygous (Exon 11: T/C and Intron 9: T/A) in both SNPs. The *PMEL17ex11 *SNP (as well as *UPP5 *and *UPP6 *also in complete linkage with the *MCOA *locus) shows a near perfect correlation between phenotype and genotype in our horse material. Genotype information on *PMEL17ex11 *is shown in the four pedigrees in Figure 3. The only discrepancy is the healthy dam discussed above (Genotyping and Linkage analysis section) with a heterozygous genotype (marked in red color in Figure 3). However, since she has produced two MCOA affected offspring, this dam is most likely a case where the position and/or size of the cysts make them extremely difficult to detect. In summary, the genotype data on *UPP5*, *PMEL17ex11 *and *UPP6 *strongly support the hypothesis that horses with the Cyst phenotype are heterozygous for the mutant allele and that horses with the MCOA phenotype are homozygous for the mutant allele.

## Discussion

In the current study we have localized the *MCOA *locus to an interval on horse chromosome 6q by linkage mapping. The locus is positioned within a 4.9 Mb interval between microsatellite marker *TKY412 *and *UPP7*. This conclusion is based on one reliable recombinant on either side of the identified region. These two recombinant offspring were genotyped for all markers used in this study and the recombination events were clearly visualized by using the CHROMPIC-option in CRIMAP [17]. The order of markers could confidently be established through the publicly available horse genome sequence generated by The Broad Institute at MIT and Harvard [19]. We used the Ensembl genome browser to assess the precise position of genotyped markers. The UCSC genome browser utilizes the initial draft assembly (EquCab1). The contig containing *PMEL17 *is not anchored to a chromosome in that version. This problem had been solved in the second assembly (EquCab2), which is used by Ensembl. By blasting the sequence adjacent to our genetic markers against the human genome sequence (Ensembl, NCBI 36 assembly) we could conclude that the order of these sequences are identical in human and horse and that the horse MCOA region corresponds to a region on human chromosome 12 (HSA12). Further, we provide molecular genetic evidence that the MCOA syndrome is in agreement with a codominant mode of inheritance. MCOA affected horses were homozygous for all three markers that were completely linked to the disease locus, horses with the Cyst phenotype were heterozygous, while healthy horses had a different set of alleles. Dosage effects are present in developmental eye disorders in other species, like for example aniridia in man and *Small eye *in mice, that are both caused by mutations in *PAX6*. *Small eye *mice that are heterozygous display iris hypoplasia whereas homozygous mice lack eyes, nasal cavities and display brain abnormalities [20]. Ewart *et al*. [9] reported a limited number of nonpenetrance cases for the Cyst phenotype. One of these healthy, predicted nonpenetrance cases was included in our analysis. We could confirm that this individual was indeed heterozygous for all markers between *UCDEQ465 *and *UPP7*, which explains the production of an MCOA offspring. The occurrence of nonpenetrance might be attributable to difficulties in detecting small cysts if situated just posterior to the iris. However, as most of the nonpenetrance cases were clustered by Ewart *et al*. [9] to a specific branch of the pedigree, the potential presence of epistatic interactions between a modifier gene and the susceptibility locus cannot be excluded.

It is possible that the causative mutation for MCOA is actually the same missense mutation in *PMEL17 *that causes the Silver coat color. However, this hypothesis is challenged by the fact that MCOA-like ocular abnormalities are not known to be present among Silver colored Icelandic horses. To investigate this further, we are arranging for a veterinary ophthalmologist to examine a number a Silver colored Icelandic horses, including one that is homozygous for the *Silver *mutation. We are not excluding *PMEL17 *as the causative gene for MCOA but since our 4.9 Mb region contains numerous genes we are planning a positional cloning approach to narrow down the region before further investigation of any one candidate. It is possible that the *Silver *locus and the *MCOA *locus are merely in linkage disequilibrium in the Rocky Mountain horse breed and that the true causative mutation is positioned in close proximity to *PMEL17*. Mutations in *PMEL17 *are known to associate with ocular abnormalities in two other species. The Zebrafish mutant *fading vision *demonstrates hypopigmentation in the retinal pigment epithelium (RPE) accompanied with vision impairment [13]. This phenotype is caused by a nonsense mutation, which expects to generate a truncated protein that lacks the terminal 355 amino acid residues. The merle patterning in dogs is associated with a SINE element insertion at the boundary of intron 10 and exon 11 in *PMEL17 *[16]. Merle homozygous Dachshunds [14] and Australian Shepherds [15] frequently have ocular defects such as microphthalmia, cataract and colobomas. These defects are similar to those of the human auditory-pigmentation disorder Waardenburg syndrome according to Clark *et al*. [16]. Both the zebrafish and the dog mutants show pigmentation defects in both the coat and in the retinal pigment epithelium (RPE). Neither of these two phenotypes is identical with the defects observed in horses with the MCOA syndrome.

MCOA in the Rocky Mountain horse clearly has some overlapping phenotypes with human Anterior Segment Dysgenesis, ASD, (Bengt Schepke pers. comm.). Clinical classifications have been difficult in humans because of the complex nature of the disease [21]. Therefore, to precisely state if any of the human disorders are homologous to equine MCOA is not a simple task. Axenfeld-Rieger syndrome, Peters anomaly, Iris hypoplasia, Primary congenital glaucoma and Aniridia in humans do all fall under the spectrum of ASD [21]. Anterior Segment Dysgenesis is genetically heterogeneous in humans. Mutations in several different genes can cause the same clinical condition. Further, the same mutation in different individuals can cause different forms of ASD [21]. Mutations in several transcription factors are known to cause human ASD, for example *PITX2*, *FOXC1*, *PAX6*, *FOXE3 *and *LXM1B *and *MAF *[22]. None of these genes are positioned in the horse MCOA interval. However, the Rocky Mountain horse could at least serve as a useful model for human cornea globosa with iris hypoplasia and congenital miosis.

Equine MCOA is generally not detectable for the untrained eye, especially in its heterozygous state, which leaves the breeders unaware of the problem. This fact, together with the close linkage to the popular Silver coat color has propagated the mutant allele in the Rocky Mountain horse population. Thus, there is a real need for identifying the causative gene and subsequently developing a genetic test to allow for informed breeding decisions.

Although linkage analysis is a powerful method for identification of chromosome regions for monogenic traits, it is limited in its capacity to identify the underlying gene. However, linkage analysis in combination with a subsequent identity-by-descent mapping strategy utilizing more distantly related and/or unrelated Rocky Mountain horses provides a better resolution. The mutation containing haplotype shared among unrelated horses are much smaller than in family materials. Detection of causative mutation(s) is facilitated even further if the study is expanded to other breeds having the same phenotype. The MCOA syndrome is present among other breeds and we plan to use these in our future fine mapping studies. Identical ocular lesions have been observed in the Kentucky Mountain Saddle Horse and the Mountain Pleasure Horse. These two breeds are closely related to the Rocky Mountain horse. The phenotype is also present in the Morgan Horse, Belgian Draft horse and in American Miniature horses [1]. Thus, future studies will involve haplotype analysis of the MCOA region in other breeds known to carry MCOA syndrome. This strategy will, of course, be most effective if no locus heterogeneity exist for MCOA.

## Conclusion

We have mapped the *MCOA *locus to ECA6q, with complete linkage to the three genetic markers; *UPP5*, *PMEL17ex11 *and *UPP6*. The locus can with high confidence be positioned to a 4.9 Mb interval between microsatellite marker *TKY412 *and *UPP7*. Moreover, our results show that the disease is in agreement with a true codominant mode of inheritance. Horses with the most severe phenotype, displaying multiple ocular abnormalities, are homozygous across the MCOA interval while the group of animals having minor clinical signs is heterozygous.

## Methods

### Pedigrees

This study included exclusively animals listed in the Rocky Mountain horse breed registrar. Seventeen paternal half-sib families where MCOA is segregating were selected for linkage analysis. To show the inheritance of MCOA in our horse material, pedigrees from four of the stallions are depicted in Figure 3. One hundred thirty-one offspring were contained within the pedigree where more than half (70 horses) of the offspring had the Cyst phenotype and about one third (38 horses) had MCOA. Out of the 94 different dams (with an average of 1.4 offspring per dam) included in the pedigree, DNA was available from 41. Out of these, eight had MCOA, 21 had the cyst phenotype and 12 were healthy. All stallions except one were predicted heterozygous for the disease i.e. had the Cyst phenotype. The one stallion was healthy and had only two offspring. The number of offspring and dams of each family, as well as the number of individuals in each phenotype category (MCOA, Cyst or healthy) per family are compiled in Table 1.

### Phenotype assessment

Assessment of phenotype was performed as described previously [1,9]. Briefly, pupillary light reflex was first assessed and further ophthalmic examination was conducted subsequent to pupillary dilation with 1% tropicamide instilled in the conjunctival fornix. Examination included direct, focal and diffuse slit lamp biomicroscopy, indirect ophthalmoscopy and applanation tonometry. The posterior segments of the eyes have been examined along with the anterior parts. The examinations revealed abnormalities of the posterior segments along with cysts in all horses we predict to be homozygous, as well as a fraction of the horses predicted to be heterozygous [9]. Data on histology for part of the horses used in this study, including findings of retinal dysplasia with changes in both the neurosensory retinal epithelium and the retinal pigmented epithelium are described in Ramsey *et al*. [1]. Blood samples were collected for DNA isolation at the time of clinical examination. The phenotyping was approved by the Michigan State University Institutional Animal Use and Care Ethics Committee.

### Genetic markers, genotyping and linkage analysis

We genotyped eleven genetic markers covering 45.2 Mb (Table 2) of ECA6q. These included five previously identified microsatellite markers and five novel microsatellites markers attained from the horse genome sequence (EquCab1 Jan. 2007) [23] by displaying tracks from simple tandem repeats recognized by Tandem Repeats Finder [24]. The SNP in exon 11 of *PMEL17 *that is associated with the Silver coat color [11] was also utilized as a marker. Primers for novel microsatellites were designed using Primer3 [25]. The PCR reactions for amplifying microsatellites were carried out separately and in a total volume of 10 μl; 40 ng genomic DNA, 2.5 mM MgCl_2_, 1× PCR Gold buffer, 0.75 units AmpliTaq Gold polymerase, 0.24 mM dNTP, 0.1 mM forward primer, 1.0 mM reverse primer, 1.0 mM labeled M13 primer. The M13 primers were labeled with FAM or TET. Amplified fragments were multiplexed when possible and separated using a MegaBACE™1000 instrument according to manufactures instructions. The result was analyzed in Genetic Profiler version 2.4 (Amersham Biosciences). SNP genotype assessment was done by pyrosequencing essentially as described in Pielberg *et al*. [26]. CRI-MAP version 2.4 [17] was used to analyze linkage between the markers and the MCOA susceptibility locus. Since the disease is codominant, it is possible to score each individuals genotype on the basis of their phenotype. A healthy horse was scored as homozygous for healthy alleles, an MCOA horse as homozygous for disease causing alleles and a horse with the Cyst phenotype as heterozygous. The TWO-POINT option was used to calculate recombination fractions (Θ) and LOD scores. BUILD was used to give the most likely position of the *MCOA *locus after positioning all markers according to the horse genome sequence [18]. The output of CHROMPIC identified unlikely double recombinants that could reveal genotyping errors.

### Sequencing of PMEL17

Primer sequences used to amplify *PMEL17 *can be found in Brunberg *et al*. [11]. Some additional novel primers were designed using Primer 3 [25], see additional file 1: Table 4. The reactions were carried out as described in [11] and results were analyzed in CodonCode Aligner version 1.6.3 (CodonCode Corporation, Dedham, MA). All eleven exons and both UTR of *PMEL17 *were sequenced. Further, all introns were fully sequenced except 1, 3, 6 and 10. Seventy base pairs (bp) were sequenced into each side of intron 1. Thirty bp were sequenced at the beginning of intron 3 and 80 bp at the end. Intron 6 was sequenced 390 and 590 bp at the start and end, respectively and intron 10 was sequenced 370 bp and 200 bp, respectively. We also sequenced approximately 370 bp upstream and 210 bp downstream of the gene.

## Authors' contributions

LSA identified and analyzed novel microsatellites including primer design, PCR and genotyping as well as genotyped a few of the previously identified markers in some of the families. LSA sequenced *PMEL17*, performed most of the linkage analysis and drafted the manuscript. RJ did initial genotyping of *PMEL17 *and collected DNA from horses used in the initial phase of the study. DTR phenotyped and collected blood samples from all horses. JE-B constructed the family pedigrees. SE initiated and designed the study, supplied the DNA along with detailed family and disease status data as well as provided valuable input and comments on the manuscript. GC initiated and designed the study, analyzed genetic markers and provided valuable input and comments on the manuscript. GL initiated and designed the study, supervised the molecular work in Uppsala, performed linkage analysis and edited and made improvements on the manuscript.

All authors read and approved the final manuscript.

## Supplementary Material

Additional File 1 **Table 4.** Primer sequences used to sequence *PMEL17*, not included in Brunberg *et al*. 2006 Click here for file
