# Supplementary material for: Equine Multiple Congenital Ocular Anomalies maps to a 4.9 megabase interval on horse chromosome 6
Source: BMC Genet. 2008 Dec 19;9:88. doi: 10.1186/1471-2156-9-88 (PMC2653074; doi:10.1186/1471-2156-9-88)
Supplement: Additional File 1 — Table 4. Primer sequences used to sequence PMEL17, not included in Brunberg et al. 2006 [file 1471-2156-9-88-S1.pdf]

Table 4 - Primer sequences for the sequencing of horse *PMEL17*, not included in Brunberg *et al.* 2006

Primer	Forward primer (5'-3')	Reverse primer (5'-3')	Probe Database
Intron 1	GGGTACTCTTCTGGCTGTGG	CACTGAGCATCCCACCACTA	Probe ID: 9710441
Upstream 1	ACTCCGTACGTGCCCTCTC	TCTCAGCTTCCTGCTCTTCG	Probe ID: 9710442
Upstream 2	AATTTGGGCTCGAGTTTCCT	CTCATTTGCATAGCCCTTCC	Probe ID: 9710443
ex11- 3' UTR	GGCGCAGACTTATGAAGCAG	TCTTTGGCTTCTTGTGCTGA	Probe ID: 9710444
